# Time-Course of Changes in Inflammatory Response after Whole-Body Cryotherapy Multi Exposures following Severe Exercise

**DOI:** 10.1371/journal.pone.0022748

**Published:** 2011-07-28

**Authors:** Hervé Pournot, François Bieuzen, Julien Louis, Jean-Robert Fillard, Etienne Barbiche, Christophe Hausswirth

**Affiliations:** 1 Research Department, National Institute of Sport, Expertise and Performance (INSEP), Paris, France; 2 Laboratory of Physiological Adaptations, Motor Performance and Health (EA 3837), Faculty of Sport Sciences of Nice-Sophia Antipolis, Nice, France; 3 Medical Department, National Institute of Sport, Expertise and Performance (INSEP), Paris, France; 4 Capbreton, France; Universidad Europea de Madrid, Spain

## Abstract

The objectives of the present investigation was to analyze the effect of two different recovery modalities on classical markers of exercise-induced muscle damage (EIMD) and inflammation obtained after a simulated trail running race. Endurance trained males (n = 11) completed two experimental trials separated by 1 month in a randomized crossover design; one trial involved passive recovery (PAS), the other a specific whole body cryotherapy (WBC) for 96 h post-exercise (repeated each day). For each trial, subjects performed a 48 min running treadmill exercise followed by PAS or WBC. The Interleukin (IL) -1 (IL-1), IL-6, IL-10, tumor necrosis factor alpha (TNF-α), protein C-reactive (CRP) and white blood cells count were measured at rest, immediately post-exercise, and at 24, 48, 72, 96 h in post-exercise recovery. A significant time effect was observed to characterize an inflammatory state (Pre vs. Post) following the exercise bout in all conditions (p<0.05). Indeed, IL-1β (Post 1 h) and CRP (Post 24 h) levels decreased and IL-1ra (Post 1 h) increased following WBC when compared to PAS. In WBC condition (p<0.05), TNF-α, IL-10 and IL-6 remain unchanged compared to PAS condition. Overall, the results indicated that the WBC was effective in reducing the inflammatory process. These results may be explained by vasoconstriction at muscular level, and both the decrease in cytokines activity pro-inflammatory, and increase in cytokines anti-inflammatory.

## Introduction

Athletes participating in competitive sports are often exposed to over-load training and competition, which may include repeated, high-intensity exercise sessions performed multiple times per week [1]. Intense training and competition particularly with under-recovery time could induce muscle damage and subsequent inflammation indicated by muscle soreness, swelling, prolonged loss of muscle function and the leakage of muscle proteins, such as C-Reactive Protein (CRP) in the circulation [2], [3]. The essential component of the physical stress theory is that high intensity physical exercise creates muscle damage and inflammation leading to disturbance in cellular homeostasis and discomfort, a phenomenon that is referred to as delayed onset muscle soreness (DOMS) [4]–[7]. In this context, the scientific interest in sports recovery modalities has been increasing in the recent years [8]. However, few studies have focused on surrogate outcomes as markers of inflammation and skeletal muscle recovery (*i.e*. leukocytes, enzymes activity, CRP) related to recovery after cold treatment following a single bout of severe exercise [7], [9], [10].

High training volumes and/or insufficient recovery has been associated with muscular fatigue, soft tissues injury, and/or immune compromise [6]. The mechanical damage to the contractile unit or plasma membrane occurs primarily due to the eccentric component of muscle movement. This insult may initiate metabolic/chemical pathways in the following hours or days, creating further damage causing an alteration in the flow, quantity and function of the immune system [2], [11], [12]. These events lead to a generalized biphasic inflammatory cascade in response to muscle damage, which involves briefly the release of various cytokines.

Strenuous exercise induces an increase in the pro-inflammatory cytokines Tumor Necrosis Factor alpha (TNF-α) and interleukin 1 Beta (IL-1β) and a dramatic increase in the inflammation responsive cytokine interleukin 6 (IL-6). This is balanced by the release of cytokine inhibitors interleukin 1 receptor alpha (IL-1ra) and the anti-inflammatory cytokine interleukin 10 (IL-10) [11]. The highest concentration of IL-6 has been found immediately after a marathon race, whereas IL-1ra peaks 1 h post-exercise (128-fold and 39-fold increases, respectively, compared to the pre-exercise values). The plasma level of IL-10 showed a 27-fold increase immediately post-exercise. The plasma level of IL-1β and TNF-α peak in the first hour after the exercise (2.1-, 2.3- fold, respectively). The pro inflammatory cytokines including IL-1β facilitate an influx of lymphocytes, neutrophils, monocytes, which participate in the healing of tissue [11], [13]. Moreover, the plasma level of C-reactive protein (CRP) increases and peaks 24 h (3-fold) after plyometric exercise or a marathon race compared to the pre-exercise value [3], [11], [13], [14]. However, inadequate or excessive inflammatory response may lead to improper cellular repair, tissue damage, and muscle dysfunction leading to loss in performance [15].

Achieving an appropriate balance between training and competition stresses and recovery is important in maximizing the performance of athletes [16]. In this context, the development of methods to speed-up the recovery of elite athletes from intense training and/or competition has been a major target of athletes and their support staff for many years [8]. Athletes, therefore, use many different therapeutic interventions, such as low intensity exercise and cold therapy (*i.e.* ice pack, shower, fan, ice ingestion, wet towel, cold water immersion (CWI)), in an effort to speed-up recovery between intense bouts of exercise or competition stress and maintain sport performance [7], [17]. Cold therapy is commonly used as a procedure to alleviate pain symptoms, particularly in inflammatory diseases, injuries and overuse symptoms and thereby aiding recovery after soft-tissue trauma [18]–[20]. Although CWI has a relative low cost, the time required for therapists to prepare CWI is time consuming. In addition, the water and ice used in CWI can only be used once, and it is relatively difficult to control the temperature during the treatment [9]. A recent method designated the whole-body cryotherapy (WBC) has been progressively used as an efficient tool in biological regeneration of healthy and physically fits individuals [17], [21]. WBC consists in a brief exposure in minimal clothing to very dry cold air (ranging from −110°C to −180°C) to the surface of the body for 2–3 min to treat the symptoms of various diseases such as arthritis, fibromyalgia and ankylosing spondylitis [18]. It already has been already demonstrated that WBC stimulated physiological reactions of an organism which result in analgesic, anti-swelling, antalgic immune and circulatory system reactions and then could improve recovery after muscle injury from muscular trauma [22]–[24]. The reported general effect of WBC suggests that it may be beneficial to sportsmen also. A recent work has shown that three repeated WBC events by the day before each training session, benefits the time it takes for the kayaker to return to full fitness and may avoid surgery [25]. The authors demonstrated that after 6 days of elite training kayakers with a mean of 4 h per day, at an extremely low temperature, was associated with a decrease of −34% in the activity of creatine kinase (CK) and a slight decrease −5% in cortisol concentration compare to the week without cryostimulation exposure [25]. Moreover, after 3 h per day of an elite rugby training program, 1 repeated WBC treatment each day over 5 days has also been shown to decrease IL-2, IL-8, CK, prostaglandin E2 (PGE2) activity, and exhibited increased concentrations of anti-inflammatory cytokines (IL-10) in peripheral blood, suggesting a local and systemic anti-inflammatory effect [10]. However, there was no precision in this study regarding when the treatment was applied before (pre-cooling) or at the end (post-cooling) of exercise. Furthermore, no case-control protocol was applied in this study and the interaction of exercise and cold exposure on immune function has not been well studied [26], [27] making it difficult to evaluate the real potential of this method of recovery.

In this context, very limited specific studies and data on inflammatory mediators are available using WBC like methods of recovery after exercise. Therefore, the primary aim of this investigation was to analyze the effect of two different recovery modalities (WBC vs. PAS) after exercise in the proposed markers for muscle damage, systemic inflammation (CRP, IL-6, IL-1β, IL-1ra, IL-10, TNF-α) and immune cell mobilization (total leukocytes, neutrophils, monocytes and lymphocytes). We hypothesized that WBC compared to PAS, accelerate the recovery in reducing exercise-induced muscle damage (EIMD) by decreasing the acute phase of inflammation in response to a single bout of exercise. A complementary aim of this study was to determine whether WBC had a positive effect on recovery from exertional muscle damage and immune function during 4 days following a single bout of exercise in well-trained runners.

## Methods

### Subjects

Eleven well-trained runners participated in the study (see Table 1 for characteristics), all with similar training levels and statures. The selected runners regularly engaged in long distance running events (e.g. marathon, trails) and presented no contraindications to cryotherapy, such as claustrophobia and cold hypersensitivity. All subjects were volunteers and were informed about the study protocol, the risks of tests and investigations, and their rights according to the Declaration of Helsinki. All subjects accepted to participate and completed the written informed consent and a health history questionnaire. The study was approved by the local Ethics Committee (Île-de-France XI, France; Ref. 200978) before its initiation.

**Table 1:** Characteristics of the study group.

Subject characteristics	Means	±	SEM
Age (years)	31.8	±	1.96
Height (cm)	179	±	1.81
Weight (kg)	70.6	±	1.96
VO_2max_ (ml. min^−^^1^. kg^−^^1^)	62	±	1.18
MAS (km.h^−^^1^)	18.7	±	0.33
10 km-run (min)	34.48	±	0.71

VO_2_max: Maximal oxygen uptake; MAS, Maximal Aerobic Speed. Values are expressed as means ± SEM of the means.

### Study Design

**Figure 1:**
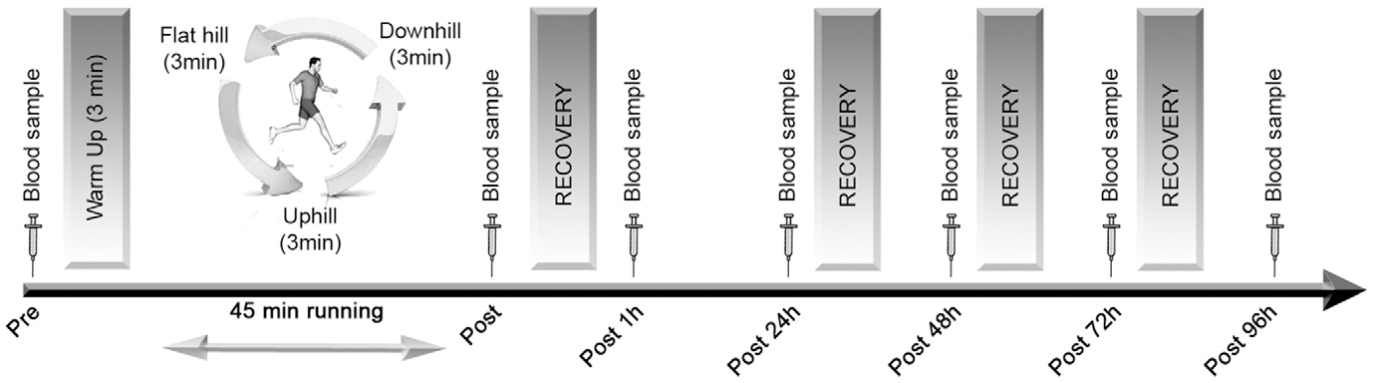
Study design - Recovery: PAS or WBC.

An overview of the experimental protocol is presented in Figure 1. All participants used both recovery modalities. Between trials, a minimum of three weeks of low intensity training was ensured. Once a month, subjects completed a simulated trail run on a treadmill followed by one of the two recovery modalities presented in a random order (WBC or PAS). Before (Pre), after the simulation (Post), after the first recovery session (Post 1 h), and before the following recovery sessions (Post 24 h, Post 48 h, Post 72 h, Post 96 h), blood samples, were collected to analyzed several markers of inflammation, muscle damage (IL-1ra, IL-1β, IL-6, IL-10, TNF-α, CRP) and the haematological profile. One week before the experiment, subjects were familiarized with the test scheme and location and preliminary testing was performed. From that week onwards until the end of the experimentation period, the training loads of all subjects were imposed and under control. The subjects refrained from consumption of any anti-inflammatory pills and did not use any additional methods to aid recovery (i.e. stretching, massage or active recovery). Participants completed food and activity diaries to standardise hydration and nutrition during the week prior to each session and no caffeine was ingested before and throughout the duration of the tests

### Preliminary Testing

One week before the experiment started, subjects came to the laboratory for a preliminary testing procedure. Maximal oxygen uptake (*,V.*O_2max_) was determined in running using a motorized treadmill (H/P/Cosmos® Saturn, Traunstein, Germany). The test consisted of a 6 min warm-up at 12 km.h^−1^ and an incremental period in which the running speed was increased by 1 km.h^−1^ every 2 min until volitional exhaustion. Oxygen uptake (*,V.*O_2_), minute ventilation (*,V.*E), and respiratory exchange ratio (RER) and respiratory rate (RR) were continuously recorded with a breath by breath gas exchange analyzer (Quark CPET, Cosmed, Roma, Italy). Heart rate (HR) was recorded using a chest belt (Cosmed wireless HR monitor, Roma, Italy). The criteria used for the determination of *,V.*O_2max_ were threefold: a plateau in *,V.*O_2_ despite an increase in power output, a RER above 1.1, and a heart rate (HR) above 90% of the predicted maximal HR [28]. *,V.*O_2max_ was determined as the average of the four highest *,V.*O_2_ values recorded (mean *,V.*O_2_max: 62.0±3.9 mL.min^−1^.kg^−1^). The first and the second ventilatory thresholds (VT1 and VT2) were determined as previously described [29]. The maximal aerobic speed (MAS) was the highest running velocity completed in 2 min (mean MAS: 18.7±1.1 km.h^−1^). Thereafter, individuals were exposed individually to a one-time session of extremely low temperature (−110°C) in a cryogenic chamber (Icelab®, Zimmer MedizinSysteme, Neu-Ulm, Germany) next to the laboratory. The session lasted 1 min. This previous familiarization session was done to check the tolerance of extremely low temperature by subjects before to start of the experiment and to accustom themselves to the cryochambers after an high intensity exercise.

### Simulated Trail Running Race

Once a month, subjects completed a simulated trail running race designed to generate fatigue, on the same treadmill used for preliminary testing. The trail run was designed to replicate as completely as possible the race constraints encountered in a trail run [30]. The race lasted 48 min and was divided in 5 blocks. The first block included 6 min on the flat (0% gradient), followed by 3 min uphill (+10% gradient) and 3 min downhill (−15% gradient). Velocity was continuously adjusted as a function of gradient in order to obtain a variety of intensities and elicit a similar metabolic demand to trail races in the field (Figure 1). Therefore, velocity at the 0% gradient was between VT1 and VT2 (mean Vflat: 15.5±0.9 km.h^−1^), while velocity at a +10% gradient corresponded to ≈80% (mean Vuphill: 11.1±0.9 km.h^−1^) of the maximal aerobic velocity at the 10% gradient [31], and velocity at −15% corresponded to velocity at VT1 (mean Vdownhill: 14.2±0.7 km.h^−1^). Blocks 2–5 consisted of 3 min at 0°, followed by 3 min uphill and 3 min downhill at the gradients and velocities previously described.

### Recovery Modalities (WBC vs. PAS)

Subjects were randomly assigned to one recuperation modality (WBC or PAS) to be used after the simulated trail running race Post, Post 24 h, Post 48 h, Post 72 h and Post 96 h. All subjects used each of the recovery modalities in the course of the experiment. WBC sessions were administered in a specially built, temperature-controlled unit (Zimmer MedizinSysteme GmbH, Ulm,, Germany), which consisted of three rooms (−10, −60 and −110°C). The temperature of all rooms remained constant throughout the experiment. During each WBC session, subjects traversed the warmer rooms and remained in the therapy room for 3 min. In the familiarization session, exposure was reduced to 1 min. Subjects were instructed to dry eventual sweat, wore a bathing suit, surgical mask, earband, triple layer gloves, dry socks and sabots. During the 3 min, subjects avoided tension by slightly moving their arms and legs by walking. After the WBC session, subjects spent 10 min seated comfortably in a temperate room (24°C) wearing a bath robe. The second recovery modality was a passive recovery (control modality) during which each subject was seated comfortably in an armchair for 30 min, in which they were not allowed to speak to anyone.

### Biochemical Analyses

To avoid inter-assay variation, all blood samples were analyzed in a single batch at the end of the study, with the exception of haematological measures, which were performed on the day of collection. Blood samples were collected from a superficial forearm vein using standard venipuncture techniques. For each blood sampling, 33 ml was directly collected into EDTA tubes (5 tubes EDTA  = 6 mL and 1 tube EDTA  = 3 mL) (Greiner Bio-one; Frickenhausen, Germany).

ENZYMATIC ANALYSES – The 5 tubes of 6 mL was centrifuged at 3000 rev.min^−1^ for 10 min, +4°C to separate plasma. The obtained plasma sample was then stored in multiple aliquots (Eppendorf type, 1500 µL per samples) at −80°C until analysis. From these samples, TNF-α, IL-6, IL-10, IL-1ra, IL-1â and CRP were determined in plasma by enzyme-linked immunosorbent assay with commercially available high sensitivity ELISA kits (R&D Systems, Minneapolis, MN, USA). All blood samples were analyzed in duplicate at respective wavelength on a spectrophotometer Dynex MRXe (Magellan Biosciences, Chelmsford, MA, USA). The sensitivity limit of CRP, TNF-á, IL-1ra, IL-1â, IL-6, IL-10 assay were respectively 0.010, 0.106, 6.26, 0.057, 0.016-0.110 (range), 0.5 pg.mL^−1^.

HEMATOLOGIC PROFILE - Blood from the 3 mL tube were analysed for leukocyte and erythrocyte count using an automated cell counter (Cell-Dyn® Ruby™, Abbott, IL, USA) by standard laboratory procedures (flow cytometry) previously described in detail [32].

### Statistical Analyses

Statistical analysis was performed using the SPSS 19 package (IBM corporation, Inc. NY, USA). We assessed the distribution of the analyzed variables using a Shapiro-Wilk test. The results showed that the distributions deviated from normal distribution, so a detailed statistical analysis using nonparametric tests was necessary: a Wilcoxon matched-pairs test was completed to assess significantly difference between groups and a Friedman rank test was undertaken to evaluate the statistical differences in time for each recovery modality. When a significant F-value in Friedmans' analysis was found, a post-hoc test with a Bonferroni correction was used to determine the between-means differences. For the parameters with normal distribution the results are expressed as the mean value with standard error of the mean (± SEM), in other cases the results are expressed as median, the value of the lower quartile (Q_25_) and the value of the upper quartile (Q_75_). The level of significance was set at p<0.05.

## Results

There were no statistically significant differences in the initial levels of any of the studied cytokines between the examined groups. These values were low and typical for healthy persons. The results obtained in subsequent samples were referred to the initial level for the group, treated as the control level.

### Enzymatic Analyses

#### Tumor Necrosis Factor-α

For both recovery modalities, there was no time effect on TNF-α and no differences between groups at anytime point (Table S1).

#### Interleukin -6 and Interleukin -10

The Wilcoxon matched-pairs test indicated no significant difference on IL-6 and IL-10 levels from post-exercise between PAS and WBC conditions. For both groups, a significant time effect (P<0.05) was observed with very similar inflammatory response regardless of recovery mode. Both IL-6 and IL-10 level increase immediately after exercise.

#### Interleukin -1β and Interleukin -1 ra

**Figure 2:**
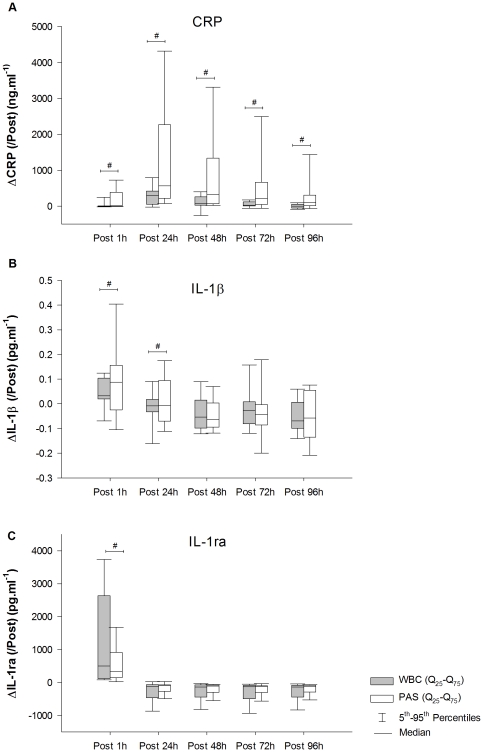
Changes in CRP (A), IL-1β (B) and IL-1ra (C) from post-running exercise to recovery. #, significant difference between groups (p<0.05). WBC, whole body cryotherapy; PAS, passive rest recovery.

The Wilcoxon matched-pairs test revealed (Figure 2B and 2C, respectively) significant differences between recovery modalities at Post 1 h (p<0.05). At Post 1 h, ΔIL-1β and ΔIL-1ra showed significant higher and lower values in the PAS condition compared to the WBC, respectively. There was also a significant (p<0.05) difference in ΔIL-1ra with lower values for the WBC condition compared to the PAS condition at Post 24 h. On raw data, the Friedman test revealed a significant difference between time measurements for both groups for each of these cytokines (P<0.05) (Table S1). Post-hoc analyses revealed that the decrease of IL-1ra occurs earlier after cryotherapy treatment than after the PAS modality (WBC: Post 24 h vs. PAS: Post 72 h). Post-hoc analysis on IL-1β revealed that plasma concentrations at Post 1 h were significantly higher (P<0.05) than Pre only for the PAS condition (Table S1).

#### Plasma C-reactive protein (CRP)

CRP level is steady whatever the condition at Post and Post 1 h compared to basal value. Analyses of Delta CRP (ΔCRP) from Post measurement showed significant (p<0.05) difference between recovery modalities at Post 1 h, 24 h, 48 h, 72 h and 96 h from exercise with significant higher values in the PAS condition compared to the WBC (Fig. 2A). A significant time effect was recorded for both groups. CRP increased (p<0.05) and peaked 24 h post-exercise in both groups (WBC = +123% vs. PAS =  +515%). In the PAS group, 48 h after exposure the increased CRP level persists (at 72 h, P = 0.052 with Bonferroni's correction) while the levels of WBC group return to the initial state.

### Leukocytes Counts

Leukocytes counts showed no significant differences (p<0.05) between modalities. The Friedman test revealed a trend towards significance between time measurements. Post-hoc analyses showed a significant increase (p<0.05) at Post 1 h of 52% and returns to the initial state by Post 24 h in both groups. Additionally, the increase concerned the number of neutrophils. There were no statistically significant changes in monocytes and lymphocytes (Table S2).

## Discussion

This study was conducted in order to analyze the effect of two different recovery modalities on classical markers of exercise-induced muscle damage (EIMD) and inflammation obtained after a simulated trail running race. We chose to compare changes in immune cell mobilisation and CRP level because they are reliable indicators of acute performance deterioration, muscle damage and/or inflammation routinely evaluated in the general population and in athletes [3], [10]. The major finding was that a single exposure to WBC significantly alleviated inflammation after a strenuous exercise run. i) Delta IL-1β was significantly suppressed 1 h after exercise following WBC, compared to the PAS condition ii) Delta IL-1ra increased 1 h and 24 h after exercise following WBC compared to PAS iii) CRP increase was strongly limited in the WBC group compared to the PAS group at 24 h and until 48 h after exercise.

Principally, trail exercise will involve substantial uphill and downhill elements. The uphill tends to result in a greater exercise intensity and hence an increased metabolic cost [33]. Conversely, downhill results in a lower metabolic cost than level and uphill walking at the same absolute speed [34], but it imposes greater forces on the lower limbs [35], resulting in greater eccentric loading. These eccentric muscle actions during downhill can result in temporary EIMD, which is manifested as reduced muscle function, muscle soreness (DOMS), efflux of intramuscular enzymes, and limb swelling that may last for several days after the exercise bout [36].

Within the injured muscle tissue there is leukocyte infiltration and local production of various pro- and anti-inflammatory cytokines which are crucial for initiating the breakdown and the subsequent removal of damaged muscle fragments [37]. As expected, the present study demonstrates that trail exercise induces a significant release and peak of IL-6 (16 fold) and IL-10 (7 fold) levels early after trail exercise compared to rest (means of both groups), followed by a rapid decrease toward pre-exercise, as demonstrated in previous studies [6], [11], [13]. However there was no significant change in the plasma concentration of the pro-inflammatory cytokine TNF-α. This lack of change was consistent with a 42 km marathon [38] and iron man race, suggesting that our population is well trained to this type of exercise [39]. Moreover, the fact that the plasma level of TNF-α was not affected immediately after the trail exercise, might explain why the monocytes were also not activated by the exercise [14]. It is also well established that high intensity exercise (>75% 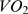
max) is associated with significant increases in circulating leukocytes (*i.e.* increases of neutrophils and falls in lymphocytes) during recovery [7]. In the present study, leukocytes increase an average of 34% above resting level. This is mainly due to an increase of the neutrophils number by 64% whereas lymphocytes felt to an average of 10% Post (mean of both groups). Furthermore, as previously described [11], high plasma concentration of IL-6 induces a peak expression of IL-1ra and IL-1β 1 h after exercise, 345% and 138%, respectively (PAS group values compared to Pre values).

Consistent with previous studies, we find similarly that increased cytokines levels were related to a significant increase and peak in CRP 24 h after exercise [3], [40]. In the present study, the CRP level of the PAS group increased 6-fold 24 h after the simulated running race compare to Pre value vs. 3 fold or 31 fold in previous studies [3], [39]. However, these differences compared to the first study might be explained by the greater muscle mass mobilized by lower limb vs. elbow or the used of eccentric activation vs. concentric actions in the previous study [3]. Secondly, unlike results with the second study cited [39] may be explained by the difference in the type and duration of exercise leading to greater acute phase response than following trail exercise. Indeed the iron man triathlon race consisted of about 10 h of exercise (swim, bike, run) vs. only 48min trail run exercise in the present study.

The amalgamation of these damaging effects can be problematic for activity on subsequent days, and there may be a greater risk of injury due to residual soreness and perturbations in muscle function [41]. This study measured the selected cytokines TNF-α, IL-6, IL-10, IL-1ra, IL-1β and CRP in well-trained athletes for up to 96 h following a trail exercise. IL-6 and IL-10 levels are not influenced by one session of WBC repeated on four consecutive days. However, contrary to previous reports suggesting that WBC exposure increased the anti-inflammatory cytokine IL-10 production [10], our results present no significant changes after 4 exposures to WBC, compared to PAS modality. Nevertheless, the different type of exercise, 3 h by day of Elite training rugby during 4 days *vs.* 48 min running exercise in the first day might explain this difference of result between studies. However this previous study did not utilize a control passive group as in the present study, in order to state that the increase in IL-10 is due to cryotherapy and not to the repetition of exercise itself. Moreover, they conducted the study on a more acute time line, 7 days vs. 5 days in the present study, which might lead for the difference of IL-10 response.

There is accumulating evidence in the literature that IL-1β is balanced by the release of cytokine inhibitors such as IL-1ra which restrict the magnitude and duration of the inflammatory response to exercise [11]. At Post 1 h, ΔIL-1ra and ΔIL-1β from Post are up-regulated and down-regulated after a single WBC session, respectively, and ΔIL-1β remain significantly different (p<0.05) at Post 24 h when compared to values taken during control passive rest recovery (Figure 2). Excepted the study of Lubkowska et al. [21] that showed changes of the IL-6 and IL-1 level, after multiple WBC exposure, literature on the cytokines cascade after exercise and the influence of WBC is very sparse and do not provide related results to both IL-1 and IL-6.

WBC is not effective in modulation of leukocytes population after 4 sessions of WBC following trail exercise. This result is in accordance with a previous study, which showed no significant changes in leukocytes count after 10 sessions of WBC, applied 2 days following progressive ergocycle test until volitional exhaustion [42]. In parallel to IL-1 modulation, neutrophils numbers were recovered 24 h after exercise in both groups. However to the best of our knowledge, there is no previous study related to neutrophils following exercise and WBC sessions. Published data suggest that WBC has no detrimental effect on immunological parameters, although the observation period in the present study may be too short to evaluate changes in monocytes, lymphocyte involvement and function [10].

A previous study presented a negligible effect of WBC on CRP [10]. However, we find that a single WBC exposure suppresses the peak increase in CRP 24 h after exercise and the difference (p<0.05) of ΔCRP with PAS group initiate at 1 h until 96 h after exercise (Figure 2 A). However, the differences in exercise type between studies as previously described might also explain the differences in results. Moreover, the lower body mass index (BMI) in the study, 21.1±1.1 kg.m^−2^ herein vs. 27.2±2.3 kg.m^−2^ for the population study in Banfi et al. (2008) [10] might lead to a different impact of cold at both skin and core levels. Indeed, some studies indicate reduced cold-induced thermogenesis, due to a high level of insulation in obesity under severe cold conditions [43], and decreased autonomic responsiveness [44]. Indeed, a stimulating effect of cold exposure was found to depend on the relationship between the decrease in core temperature, and the duration of exposure [45].

In the present study, using a single exposure in WBC is associated Post 1 h with a significant decrease (p<0.05) of the pro-inflammatory mediator IL-1β (Figure 2 B) and an increase of the anti-inflammatory cytokine IL-1ra (Figure 2 C) compared to PAS. In accordance with the present results, it was shown that prolonged cold-wet (5°C) exposure following strenuous exercise also differentially modulated cytokine production, up regulating (12±3.7%) IL-1ra production and down regulating (1.1±0.05%) IL-1β secretion [46]. Moreover consistent with a previous report using cold-pack application, WBC exposure immediately after exercise had no effect on IL-6 levels and was associated with a significant decrease of IL-1β [8]. In contrast, previous study associated exercise with ice application recovery showed a significant decrease (29%) in the anti-inflammatory marker IL-1ra compare to the pre-exercise value [8]. The discrepancy for the differences in cytokine responses between studies is likely due to the nature of exercise and the aim of the method of cold exposure (*i.e.* decrease skin temperature or core body temperature) [8], [10], [46]. Cryotherapy exposure causes a drive to maintain core body temperature, resulting in local vasoconstriction [47]. In this case, the skin temperature would be a determining factor in the shortening or relaxing rate of smooth muscle in the vessel wall [48]. It has been suggested that the vasoconstriction resulting from cold exposure may result in a redistribution of blood flow away from the skin towards the muscle and core. However, data of a recent study showed that more blood was distributed to the skin in cold water [49]. This suggests that colder temperatures may be associated with reduced muscle blood flow, which could provide an explanation for the benefits of cold in alleviating exercise-induced muscle damage in sports and athletic contexts [49]. In addition, during a severe cold exposure, such as WBC, skin temperature decreases quickly due to vasoconstriction and direct skin cooling, most markedly in the extremities [50]. Indeed, this previous study showed that skin temperature recorded in the calf was 9.04±3.78°C immediately after WBC [51]. Thus WBC −110°C might induce a greater fluid shift than other method, which accelerates turn-over process.

In general, we observed an exercise induced neutrophilia in all trials (Table S2). During recovery after WBC, circulating neutrophil counts increased by an average of 114% above baseline value, with the largest increase 1 h after exercise. In contrast, the average increase in neutrophil counts was lower during PAS (101%). In accord with the result of a previous study, acute cold stress increased significantly circulating neutrophil counts [7], [52]. In the literature, neutrophils depletion significantly impaired their angiogenic function (via the vascular endothelial growth factor (VEGF)) [53]. This adaptive change (angiogenesis) is one of the physiological adaptations for the improvement of perfusion, physical performance and other health benefits [54]. Thus, WBC might contribute to angiogenesis, and decrease DOMS and time of recovery.

Limited evidence suggests that cold exposure may also initiate changes in cytokine expression associated with a nonspecific acute phase reaction [27]. Downstream of the change in cytokine levels, especially IL-1, we observed in this study a concomitant down-regulation of CRP when athletes used the WBC treatments. Indeed, in a previous study, the correlation between IL-1 and CRP release was stronger than that IL-6 and CRP suggesting that IL-1β is probably the more powerful stimulant of CRP release [55] Contrary to previous studies, we observe a significant decrease in CRP after WBC compared to PAS, while others have indicated negligible changes after WBC or CWI [10], [56]. Nevertheless, in both studies there is no assessment 24 h post-exercise attesting a significant increase or control group to observe any significant difference. Second, for Halson et al. (2008) [56], 1 min of exposure repeated 3 times to cold temperature of 11.5°C during the CWI method seems to be limited to induce sufficient physiological changes [57].

The mechanism underlying the abovementioned differences in cytokine generation is not clear, but it can be argued that cold-associated modulation of cytokine production may be provoked by alterations in central hemodynamics associated with enhanced thermoregulatory demands and therefore may influence immune homeostasis in cold environments [27], [46]. Since recently the direct effect of cytokines on neuroendocrine axes has been demonstrated [58]. Inflammation and immunity are under the control of many different systems, including the nervous, the endocrine and the vascular systems. Nerve endings release norepinephrine in the tissues [58]. Cold-induced vasoconstriction should be related to the reflex sympathetic activity and its attendant increase in the affinity of α-adrenoceptors in the vascular wall for norepinephrine (not measured in the present study) [59]. It binds α and β-adrenergic receptors expressed on immune cells. Moreover, a previous study demonstrated that norepinephrine was the only hormone that responded positively to WBC treatment (i.e. three time exposure, over one week) and that the sustained norepinephrine could have a role in pain alleviation (DOMS) [60]. Thus, another hypothesis has been formulated to explain the cytokines modulation. The findings of the present study (Table S1) are consistent with investigations indicating that adrenergic/noradrenergic mechanisms are intimately involved in the regulation of cytokines production with physical stress [61]. The stimulation of β-adrenoceptors during stress attenuates excessive synthesis of pro-inflammatory cytokines (IL-1β and TNF-α), and elevates anti-inflammatory cytokines (IL-6, IL-1ra and IL-10) [62]. In this context, the current observations showing that cold exposure suppressed IL-1β but stimulated IL-1ra expression, indicating that β-adrenergic mechanisms may have predominated when cold stress was preceded by exercise. This confirmed that the treatment induced an anti-inflammatory protection [10].

In conclusion, a unique session of WBC (3 min at −110°C) performed immediately after exercise enhanced muscular recovery by restricting the inflammatory process. These findings suggest that multiple interactions between cytokines are likely involved in the physiological response to exertional fatigue and cold may serve to limit the severity of the host inflammatory response. In this case, accordingly with our hypothesis, multiple WBC exposures can enhance recovery, by decreasing the acute phase inflammatory response after a running trail exercise, thus contributing to its beneficial role in organ protection after muscle damage. The present study suggests that soluble receptor antagonist IL-1ra increases after a single whole body cryostimulation (−110°C) and restrict the inflammatory response to exercise by decrease in the magnitude of IL-1β and CRP. In term of practical applications, data confirm that the treatment induces an anti-inflammatory protection effect, and suggest that WBC reduce the time of recovery by positive effects on immunological parameters and the regeneration process.

## Supporting Information

Table S1 Time course changes in cytokines before and after exercise following WBC or PAS.
(DOCX)

Table S2 Leukocytes count before and after exercise following WBC or PAS.
(DOCX)
