# Correction: Time-Course of Changes in Inflammatory Response after Whole-Body Cryotherapy Multi Exposures following Severe Exercise

**DOI:** 10.1371/annotation/0adb3312-7d2b-459c-97f7-a09cfecf5881

**Published:** 2011-11-09

**Authors:** Hervé Pournot, François Bieuzen, Julien Louis, Rémi Mounier, Jean-Robert Fillard, Etienne Barbiche, Christophe Hausswirth

The authors wish to add Rémi Mounier as the 4th author in the byline. The updated byline is: Hervé Pournot1,2, François Bieuzen1*, Julien Louis2, Rémi Mounier4, Jean-Robert Fillard3, Etienne Barbiche5, Christophe Hausswirth1

The updated Affiliations are: 1 Research Department, National Institute of Sport, Expertise and Performance (INSEP), Paris, France, 2 Laboratory of Physiological Adaptations, Motor Performance and Health (EA 3837), Faculty of Sport Sciences of Nice-Sophia Antipolis, Nice, France, 3 Medical Department, National Institute of Sport, Expertise and Performance (INSEP), Paris, France, 4 Capbreton, France, 5 Inserm U1016, Institut Cochin, Paris, France

The updated citation is: Pournot H, Bieuzen F, Louis J, Mounier R, Fillard J-R, et al. (2011) Time-Course of Changes in Inflammatory Response after Whole-Body Cryotherapy Multi Exposures following Severe Exercise. PLoS ONE 6(7): e22748. doi:10.1371/journal.pone.0022748

In addition to the other authors's contributions, Rémi Mounier's roles were: Conceived and designed the experiments, Performed the experiments, Analyzed the data, Contributed reagents/materials/analysis tools, and Wrote the manuscript 

