# Supplementary material for: Time-Course of Changes in Inflammatory Response after Whole-Body Cryotherapy Multi Exposures following Severe Exercise
Source: PLoS One. 2011 Jul 28;6(7):e22748. doi: 10.1371/journal.pone.0022748 (PMC3145670; doi:10.1371/journal.pone.0022748)
Supplement: Table S1 — Time course changes in cytokines before and after exercise following WBC or PAS. (DOCX) [file pone.0022748.s001.docx]

**Supplementary Table S1: Time course changes in cytokines before and after exercise following WBC or PAS.**

|  |  | Median and the value of the lower and the upper quartile (Q_25_-Q_75_) | | | | | | | | | | | | | | | | | | | | |
| --- | --- | --- | --- | --- | --- | --- | --- | --- | --- | --- | --- | --- | --- | --- | --- | --- | --- | --- | --- | --- | --- | --- |
|  |  | Pre | | | Post | | | Post 1h | | | Post 24h | | | Post 48h | | | Post 72h | | | Post 96h | | |
|  |  |  |  |  |  |  |  |  |  |  |  |  |  |  |  |  |  |  |  |  |  |  |
| IL6 (pg.ml^-1^) | WBC ^$^ | 0.115 | | | 3.203 * | | | 1.326 * | | | 0.063 ^#^ | | | 0.069 ^#^ | | | 0.069 ^#^ | | | 0.005 ^#^ | | |
|  |  | (0.000 | - | 0.316) | (2.152 | - | 4.767) | (0.921 | - | 2.021) | (0.000 | - | 0.518) | (0.000 | - | 0.345) | (0.000 | - | 0.589) | (0.000 | - | 0.320) |
|  | PAS ^$^ | 0.126 | | | 3.202 * | | | 1.471 * | | | 0.152 ^#^ | | | 0.190 ^#^ | | | 0.126 ^#^ | | | 0.295 ^#^ | | |
|  |  | (0.000 | - | 0.423) | (2.488 | - | 4.971) | (1.175 | - | 2.108) | (0.115 | - | 0.486) | (0.071 | - | 0.257) | (0.040 | - | 0.510) | (0.000 | - | 0.515) |
|  |  |  |  |  |  |  |  |  |  |  |  |  |  |  |  |  |  |  |  |  |  |  |
| IL10 (pg.ml^-1^) | WBC ^$^ | 0.509 | | | 6.792 * | | | 5.204 | | | 0.801 ^#^ | | | 0.743 ^#^ | | | 0.582 ^#^ | | | 0.502 ^#^ | | |
|  |  | (0.305 | - | 2.141) | (2.624 | - | 9.285) | (1.530 | - | 7.171) | (0.329 | - | 1.874) | (0.266 | - | 1.288) | (0.319 | - | 0.843) | (0.282 | - | 0.739) |
|  | PAS ^$^ | 0.651 | | | 7.434 * | | | 3.367 | | | 0.541 ^#^ | | | 0.601 ^#^ | | | 0.504 ^#^ | | | 0.430 ^#^ | | |
|  |  | (0.345 | - | 1.013) | (2.906 | - | 9.074) | (1.519 | - | 6.324) | (0.502 | - | 1.425) | (0.279 | - | 1.297) | (0.242 | - | 1.645) | (0.274 | - | 1.042) |
|  |  |  |  |  |  |  |  |  |  |  |  |  |  |  |  |  |  |  |  |  |  |  |
| IL1ra (pg.ml^-1^) | WBC ^$^ | 187 | | | 345 | | | 714 * | | | 179 ^#^ | | | 179 ^#^ | | | 148 ^#^ | | | 172 ^#^ | | |
|  |  | (122 | - | 284) | (190 | - | 657) | (527 | - | 2741) | (120 | - | 290) | (120 | - | 306) | (105 | - | 231) | (93 | - | 242) |
|  | PAS ^$^ | 231 | | | 305 | | | 709 * | | | 215 | | | 203 | | | 196 ^#^ | | | 189 | | |
|  |  | (134 | - | 254) | (237 | - | 441) | (383 | - | 1077) | (154 | - | 284) | (143 | - | 242) | (123 | - | 244) | (134 | - | 238) |
|  |  |  |  |  |  |  |  |  |  |  |  |  |  |  |  |  |  |  |  |  |  |  |
| IL1β (pg.ml^-1^) | WBC ^$^ | 0.145 | | | 0.253 | | | 0.295 | | | 0.260 | | | 0.171 | | | 0.166 | | | 0.149 | | |
|  |  | (0.132 | - | 0.223) | (0.165 | - | 0.277) | (0.244 | - | 0.347) | (0.169 | - | 0.277) | (0.131 | - | 0.196) | (0.134 | - | 0.253) | (0.127 | - | 0.167) |
|  | PAS ^$^ | 0.153 | | | 0.183 | | | 0.325 * | | | 0.276 | | | 0.183 | | | 0.141 | | | 0.197 | | |
|  |  | (0.134 | - | 0.193) | (0.169 | - | 0.281) | (0.240 | - | 0.441) | (0.184 | - | 0.309) | (0.172 | - | 0.243) | (0.125 | - | 0.274) | (0.139 | - | 0.226) |
|  |  |  |  |  |  |  |  |  |  |  |  |  |  |  |  |  |  |  |  |  |  |  |
| CRP (ng.ml^-1^) | WBC ^$^ | 176 | | | 176 | | | 183 | | | 530 *^, #^ | | | 262 | | | 164 | | | 145 | | |
|  |  | (105 | - | 265) | (150 | - | 278) | (153 | - | 414) | (339 | - | 810) | (179 | - | 552) | (148 | - | 427) | (107 | - | 340) |
|  | PAS ^$^ | 173 | | | 243 | | | 306 | | | 847 *^, #^ | | | 477 *^, #^ | | | 448 * | | | 321 | | |
|  |  | (101 | - | 290) | (107 | - | 307) | (120 | - | 590) | (514 | - | 2046) | (323 | - | 1472) | (250 | - | 909) | (162 | - | 629) |
|  |  |  |  |  |  |  |  |  |  |  |  |  |  |  |  |  |  |  |  |  |  |  |
| TNFα (pg.ml^-1^) | WBC | 0.423 | | | 0.475 | | | 1.246 | | | 0.336 | | | 0.380 | | | 0.221 | | | 0.080 | | |
|  |  | (0.023 | - | 0.701) | (0.649 | - | 1.577) | (0.369 | - | 0.742) | (0.051 | - | 0.661) | (0.108 | - | 0.980) | (0.099 | - | 0.347) | (0.051 | - | 0.101) |
|  | PAS | 0.532 | | | 0.478 | | | 0.754 | | | 0.746 | | | 0.342 | | | 0.388 | | | 0.476 | | |
|  |  | (0.242 | - | 0.760) | (0.233 | - | 0.779) | (0.655 | - | 1.086) | (0.410 | - | 1.002) | (0.288 | - | 0.383) | (0.000 | - | 0.478) | (0.042 | - | 1.117) |
|  |  |  |  |  |  |  |  |  |  |  |  |  |  |  |  |  |  |  |  |  |  |  |

^$^, represent a significant (p<0.05) time effect; *, represent a significant (p<0.05) difference from Pre; ^#^, represent a significant (p<0.05) difference from Post. All significant results were not pointed except from Pre and Post to avoid overloading the table.WBC, whole body cryotherapy; PAS, passive rest recovery.
