# Supplementary material for: Time-Course of Changes in Inflammatory Response after Whole-Body Cryotherapy Multi Exposures following Severe Exercise
Source: PLoS One. 2011 Jul 28;6(7):e22748. doi: 10.1371/journal.pone.0022748 (PMC3145670; doi:10.1371/journal.pone.0022748)
Supplement: Table S2 — Leukocytes count before and after exercise following WBC or PAS. (DOCX) [file pone.0022748.s002.docx]

**Supplementary Table S2: Leukocytes count before and after exercise following WBC or PAS.**

| PARAMETERS (numbers/mm³) | | Median and the value of the lower and the upper quartile (Q_25_-Q_75_) | | | | | | | | | | | | | | | | | | | | |
| --- | --- | --- | --- | --- | --- | --- | --- | --- | --- | --- | --- | --- | --- | --- | --- | --- | --- | --- | --- | --- | --- | --- |
|  |  | Pre | | | Post | | | Post 1h | | | Post 24h | | | Post 48h | | | Post 72h | | | Post 96h | | |
|  |  |  |  |  |  |  |  |  |  |  |  |  |  |  |  |  |  |  |  |  |  |  |
| Leukocytes | WBC ^$^ | 5000 | | | 6600 | | | 8000 * | | | 5500 | | | 5200 | | | 5300 | | | 5200 | | |
|  |  | (4725 | - | 6100) | (6200 | - | 7550) | (7600 | - | 9200) | (5100 | - | 6100) | (4600 | - | 6100) | (4600 | - | 5850) | (4450 | - | 6400) |
|  | PAS ^$^ | 5600 | | | 6800 | | | 7800 * | | | 5200 | | | 5300 | | | 5200 | | | 5700 | | |
|  |  | (4400 | - | 6000) | (5600 | - | 9100) | (6000 | - | 9200) | (4900 | - | 6000) | (4600 | - | 5700) | (4800 | - | 6300) | (5100 | - | 6700) |
|  |  |  |  |  |  |  |  |  |  |  |  |  |  |  |  |  |  |  |  |  |  |  |
| Neutrophils | WBC | 2988 | | | 4548 | | | 6680 * | | | 2996 | | | 2896 | | | 2754 | | | 2932 | | |
|  |  | (2813 | - | 3325) | (4124 | - | 5223) | (5691 | - | 7268) | (2739 | - | 3588) | (2538 | - | 3261) | (2652 | - | 4071) | (2370 | - | 3889) |
|  | PAS | 2979 | | | 4680 | | | 5670 * | | | 2790 | | | 2907 | | | 3024 | | | 3364 | | |
|  |  | (2296 | - | 3248) | (3976 | - | 5460) | (4440 | - | 7068) | (2244 | - | 3240) | (2350 | - | 3132) | (2704 | - | 3654) | (2907 | - | 3894) |
|  |  |  |  |  |  |  |  |  |  |  |  |  |  |  |  |  |  |  |  |  |  |  |
| Lymphocytes | WBC ^$^ | 1632 | | | 1392 | | | 1232 | | | 1848 | | | 1608 | | | 1617 | | | 1656 | | |
|  |  | (1374 | - | 1984) | (1225 | - | 1735) | (1036 | - | 1545) | (1624 | - | 2107) | (1415 | - | 1998) | (1380 | - | 1980) | (1392 | - | 1836) |
|  | PAS ^$^ | 1806 | | | 1536 | | | 1326 | | | 1850 | | | 1800 | | | 1800 | | | 1710 | | |
|  |  | (1488 | - | 1976) | (1428 | - | 1863) | (1104 | - | 1440) | (1560 | - | 1974) | (1242 | - | 1880) | (1488 | - | 2080) | (1593 | - | 1856) |
|  |  |  |  |  |  |  |  |  |  |  |  |  |  |  |  |  |  |  |  |  |  |  |
| Monocytes | WBC | 346 | | | 348 | | | 468 | | | 384 | | | 384 | | | 384 | | | 364 | | |
|  |  | (313 | - | 384) | (300 | - | 377) | (358 | - | 507) | (329 | - | 488) | (322 | - | 419) | (309 | - | 477) | (327 | - | 420) |
|  | PAS | 404 | | | 357 | | | 460 | | | 378 | | | 354 | | | 364 | | | 364 | | |
|  |  | (334 | - | 550) | (315 | - | 495) | (420 | - | 496) | (324 | - | 490) | (322 | - | 424) | (315 | - | 441) | (330 | - | 464) |
|  |  |  |  |  |  |  |  |  |  |  |  |  |  |  |  |  |  |  |  |  |  |  |

^$^, represent a significant (p<0.05) time effect; *, represent a significant (p<0.05) difference from Pre;. All significant results were not pointed except from Pre to avoid overloading the table. WBC, whole body cryotherapy; PAS, passive rest recovery.
